# Construction of multiple concentration gradients for single-cell level drug screening

**DOI:** 10.1038/s41378-023-00516-0

**Published:** 2023-04-13

**Authors:** Shaofei Shen, Fangjuan Zhang, Yali Zhang, Yi Li, Yanbing Niu, Long Pang, Jinyi Wang

**Affiliations:** 1 Shanxi Key Lab for Modernization of TCVM, College of Life Science, Shanxi Agricultural University, Taigu, Shanxi 030801 China; 2 School of Basic Medical Science, Xi’an Medical University, Xi’an, Shaanxi 710021 China; 3 College of Chemistry and Pharmacy, Northwest A&F University, Yangling, Shaanxi 712100 China

**Keywords:** Chemistry, Engineering

## Abstract

Isolation and manipulation of single cells play a crucial role in drug screening. However, previously reported single-cell drug screening lacked multiple-dose concentration gradient studies, which limits their ability to predict drug performance accurately. To solve this problem, we constructed a multiconcentration gradient generator in which a Tai Chi-spiral mixer can accelerate solution mixing in a short time and produce a linear concentration gradient. Later, a gradient generator combined with a single-cell capture array was adopted to investigate the effects of single or combined doses of 5-fluorouracil and cisplatin on human hepatoma cells and human breast carcinoma cells (at the single-cell level). The results showed that both drugs were effective in inhibiting the growth of cancer cells, and the combination was more effective for human hepatoma cells. In addition, the relationship between the biomechanical heterogeneity (e.g., deformability and size) of tumor cells and potential drug resistance at the single-cell level was investigated, indicating that small and/or deformable cells were more resistant than large and/or less deformable cells. The device provides a simple and reliable platform for studying the optimal dosage of different drug candidates at the single-cell level and effectively screening single-agent chemotherapy regimens and combination therapies.

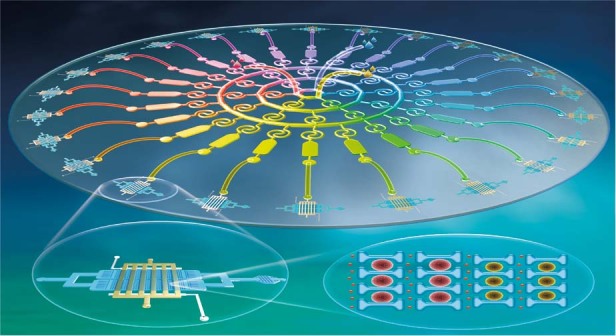

## Introduction

The rapid development of drug screening techniques presents a viable solution to preventing infections and treating human diseases^1^. Due to patient participation in preclinical validation and clinical trials, new drug development has become a costly, risky, and time-consuming project^2–4^. The pharmaceutical industry is confronted with many difficulties, including escalating costs and protracted timelines for the development of new drugs^5–7^. Additionally, in genetically identical populations, the heterogeneity of individual cells is crucial for cell growth and development. Phenotypic heterogeneity among genetically identical cells plays a crucial role in tumor metastasis, drug resistance, and stem cell differentiation^8–10^. Single-cell isolation and manipulation strategies are of considerable importance in revealing cell heterogeneity, disease diagnosis, drug delivery, and cancer biology^11–13^. Therefore, it is essential to thoroughly analyze single cells, from their survival state to their lysis state^14^. To fully understand the heterogeneity of cells, it is necessary to use traditional biological tools, such as Petri dishes and porous plates, to perform diversified operations and comprehensive analysis of cells at the single-cell level. However, there exist many difficulties and challenges in the process and evaluation of single cells of small size^15–17^. Multicomponent, high-sensitivity detection and high-throughput analysis of a large number of individual cells remain key challenges in achieving this goal.

As one of the most representative microanalysis platforms in this century, microfluidic chip technology has become a popular research topic because of its advantages of low reagent consumption, integrality, easy control, and good biocompatibility^11,18^. Among microfluidic platforms, microfluid-based systems for single-cell studies offer a powerful approach as the study of cell population heterogeneity progresses^19,20^. Compared with traditional petri dish or orifice plate experiments, studies on microfluidic single cells offer many advantages, such as high throughput, small sample size, automatic sample processing, and low contamination risk, making microfluidics an ideal technology for single-cell analysis to reveal key information about cellular heterogeneity that is often obscured in traditional ensemble measurements^11,17^.

Microfluidic chips are a powerful tool for gradient generation due to their advantages of high throughput and low consumption^21–23^. These systems have been developed for reagent mixing and drug screening by way of producing different species concentrations without manual pipetting^24–26^. Drug screening and treatment optimization otherwise can require the study of dose-dependent cellular responses at different drug concentrations, so concentration-gradient microfluidic chips have become a powerful tool in this field^27–29^. Their miniature size allows for parallelization with a minimal sample requirement, which is critical for high-throughput drug screening^30–32^. In addition, concentration-gradient microfluidic chips can be used for quantitative and large-scale assessments of toxicity and optimal concentrations of different drugs, which can not only improve the throughput and reduce the experimental cost but also rapidly and accurately control and interact with gradients at a higher resolution^33,34^. At present, microfluidic chips mostly use double or multiple-concentration gradients to study the entire cell population, conditions which cannot simultaneously study the single effect and interaction of two drugs on tumor cell heterogeneity at the single-cell level^35,36^. This paradigm may lead to a misunderstanding of tumor cell heterogeneity and the loss of key information. The establishment of multifunctional, single-cell drug screening integrated microfluidics systems based on multiconcentration gradients to predict drug synergies and optimal dosages professionally remains an urgent and challenging task. It is of great significance to provide guidance for determining rational drug combinations in clinical applications.

Our previous work has proven that the unique inertial microfluidic method can effectively reduce flow dependence and quickly build a stable and controllable multiconcentration gradient microfluidic device^37^. After, a device was also established for multistage microfluidics that was used for the isolation and capture of single cells based on cell size and deformability^38,39^. Based on this, we optimized the microfluidic device to achieve the formation of a higher flux concentration gradient, and by combining it with a single-cell capture array, we successfully constructed a single-cell microfluidic drug screening platform. In this study, the chemical gradients produced by a concentration-gradient generator were theoretically processed, calculated, and verified in a fluorescence experiment. Subsequently, cisplatin and 5-fluorouracil were used as model drugs to perform single or multidrug combination chemotherapy on human breast carcinoma (MCF-7) cells and human hepatoma (HepG2) cells at the single-cell level. Then, the applications of the system in gradient construction, single-cell capture, cell culture, and single-cell analysis were demonstrated. The results show that the developed device can explore the heterogeneity of tumor cells under multiple drug gradients with the required stability and high-throughput capability. We propose that this system provides a flexible and controllable platform for the study of pharmacological functions and other fields involving concentration gradients and single-cell analytical operations.

## Materials and methods

### Device fabrication

The microfluidic devices used in this study were obtained by soft etching with an AZ 50XT master mold on a silicon substrate. First, the microdevice structure was designed in AutoCAD software. Second, the above elements were printed on the transparent film to form a chrome mask (MicroCAD Photomask, Ltd., Suzhou, China). Finally, the mold was fabricated under UV light using AZ 50XT photoresist on the BG401A mask allocator (7 mW cm^−2^, CETC, China). Before the fabrication of our microfluidic device, the mold was exposed to trimethylchlorosilane vapor for 3 min. Fully mixed RTV 615 polydimethylsiloxane (PDMS) and curing agent (10:1, w/w) were poured onto the mold and placed in a Petri dish to yield a 3 mm-thick PDMS replica. After degassing, the mold was placed at 80 °C for 50 min, and then the PDMS replicas were stripped from the mold. The PDMS replicas were punched with a metal pin for construction treatment and backup. Afterward, the PDMS replica was trimmed, cleaned, and placed on a clean glass slide (3000 rpm, 60 s, ramp 15 s) with a PDMS prepolymer [RTV 615 A and B (20:1, w/w)] and cured for 20 min in an oven at 80 °C. Finally, the microfluidic device was ready for use after baking at 80 °C for 48 h.

### Numerical simulation

To evaluate fluid motion in the microfluidic system, computational fluid dynamics (CFD) simulation was performed using ESI-CFD software (V2010.0, ESI-CFD, Inc., Huntsville, AL, USA). Different flow rates were specified at the input, and the outlet was set to a fixed pressure boundary condition. No slip boundary condition was applied at the channel walls. FLOW and CHEM modules in CFD-ACE+ were used to explore fluid phenomena in the microchannels. Based on the finite volume method, the conservation of Navier–Stokes momentum in the device is described by Eq. (1) as follows:

1 $$\frac{\partial }{{\partial {{{\mathrm{t}}}}}}\left( {\rho \mathop{V}\limits^{\rightharpoonup} } \right) + \nabla \cdot \left( {\rho \mathop{V}\limits^{\rightharpoonup} \mathop{V}\limits^{\rightharpoonup} } \right) = - \nabla P + \overline{\overline \tau }$$

The conservation of mass is described by the continuity equation, Eq. (2), as follows:

2 $$\frac{{\partial \rho }}{{\partial t}} + \nabla \cdot \left( {\rho \mathop{V}\limits^{\rightharpoonup} } \right) = 0$$

where *ρ* is the fluid density, $\mathop{V}\limits^{\rightharpoonup}$ is the velocity vector, *P* is the pressure, $\overline{\overline \tau }$ is the stress tensor, *t* is the time, and ∇ is the standard spatial grad operator. The physical properties of water were applied to the fluids participating in the simulation (density *ρ* = 1000 kg m^−3^ and dynamic viscosity *μ* = 10^−3^ kg m^−1^ s^−1^). A diffusion coefficient *D* = 10^−10^ m^2^ s^−1^ was used for the fluids in the simulations. In addition, for fluid mixing calculation, water A and B are set as 0 and 1, respectively. A second-order limiting scheme was used to solve the species diffusion. The convergence limit for the mass fraction was set to 10^−6,^ and the simulations were run for ~2000 time steps until the flow reached the outlet.

### Experimental setup

During each test, a 1 mL one-use syringe was operated to load prepared samples as described in the Supplementary Information. Three Longer® syringe pumps (LSP04-4A, China) were utilized to regulate the operated flow rates. A 25 cm long Tygon soft tubing with an inner diameter of 0.42 mm (Longer pump, China) was used for connection. Prior to use, the microchip was initially irradiated with UV light for 2 h and then sequentially rinsed with 70% ethanol for 2–3 min to eliminate air bubbles at a flow rate of 10 μL ∙ min^−1^, followed by rinsing in ultrapurified water and PBS working buffer. These same flow rates were introduced to three inlets during process testing.

### Cell capture and treatment

Two types of human cancer cell lines, namely, MCF-7 cells and HepG2 cells, were used to investigate and analyze the feasibility of our system. MCF-7 cells and HepG2 cells were cultured in Dulbecco’s modified Eagle medium (DMEM) supplemented with 10% phosphate-buffered saline (PBS) using standard techniques, with 100 U mL^−1^ penicillin and 100 μg mL^−1^ streptomycin. These cells were then grown and maintained in a humidified atmosphere with 5% CO_2_ at 37 °C and were normally passaged at a ratio of 1:3 every three days to maintain their exponential growth phase. They were harvested through trypsinization with 0.25% trypsin (Invitrogen) in Ca^2+-^ and Mg^2+-^free Hank’s balanced salt solution at 37 °C before use. Trypsinization was stopped upon the addition of fresh supplemented DMEM, and the cell suspension was centrifuged at 1000 rpm for 2 min and then diluted to the required concentrations with DMEM.

To assess the efficacy of 5-fluorouracil (5-FU) and cisplatin (DDP) in our cell culture models, cells were plated and grown to 50–70% confluence prior to treatment with 5-FU and DDP at increasing concentrations that are in accordance with concentrations formed by the microfluidic device. Subsequent to treatment, cells were tested for the efficacy of 5-FU and DDP, as described below. All cells were cultured for ≤ 4 months before being discarded, and fresh, frozen cells were used to continue studies.

For single-cell capture devices in the microfluidic chip, the devices were initially sterilized using sequential rinsing, i.e., by initially flushing 75% ethanol, followed by Millipore ultrapure water, PBS (0.01 M, pH 7.4) and fresh DMEM and then treated with a sterilized 50 μg ∙ ml^−1^ fibronectin (FN) solution for 2 h at 37 °C to promote cell adherence, followed by cell seeding at a cell density of 1.0 × 10^5^ cells mL^−1^ at a flow rate of 200 μL/min. The surface of PDMS did not contribute to the adherent growth of tumor cells. Extracellular matrix FN solution was chosen to achieve PDMS surface modification. We introduced FN solution into every chamber and promoted the adsorption of FN to the surface of the device in a humidified atmosphere at 37 °C for 2 h. Then, serum-free DMEM was used to soak the chambers for 1 h, and the excess protein was removed. DMEM with 5-FU (100 μM), DMEM with DDP (10 μM) and drug-free DMEM were continuously injected into the inlets through the pump to investigate their efficacy on the tumor cells. MCF-7 cells and HepG2 cells were seeded in 24 single-cell capture devices. Finally, the cells were stimulated by the drug, and cell viability was detected after 2 h.

### Microscopy and image analysis

All experiments were executed on the objective table of the Olympus^®^ inverted fluorescence microscope (CKX41, Japan) as detailed in the Supplementary Information. Bright-field and fluorescence microscopy images were captured by an equipped charge-coupled device camera (Olympus, DP72, Japan) with constant imaging parameters. All captured images were processed and analyzed by Image-Pro^®^ Plus 6.0 (Media Cybernetics, Silver Spring, USA) software. Origin 9 (Origin Inc., USA was utilized for further data analysis.

## Results and discussion

### Design of the microfluidic device

**Fig. 1:**
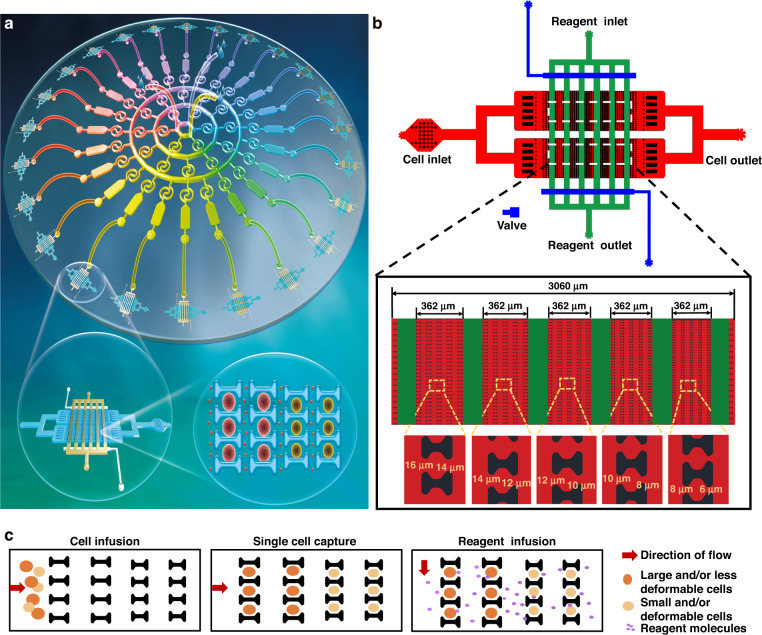
Drug screening at the single-cell level based on multiconcentration gradient construction and a single-cell capture device. **a** Schematic diagram of the integrated microfluidic device. **b** The detailed design of a single-cell capture device. The various channels are shown with different colors to visualize the microfluidic device’s different components. Red and green indicate the fluidic channels of cells and reagents, respectively, and blue shows the control channels and valves. **c** Schematic of manipulation of single-cell capture according to cell size and deformability. The procedure consists of three steps: cell infusion, single-cell capture, and reagent infusion

As shown in Fig. 1a, we designed and constructed a microfluidic operation platform, which was mainly composed of a concentration-gradient generator and 24 single-cell capture devices. The concentration-gradient generator consists of a fluid layer, a thin PDMS layer, and a glass slide. The flow layer consists of 42 Tai Chi-spiral mixers (50 μm width, 50 μm height), 24 liquid storage chambers (1200 μm width, 2200 μm length, 50 μm height), three sample intakes, and 24 exits (Fig. S1). Three sample entrances were used to fill the source solution. Spiral micromixers were mainly used to diffuse and mix solutions from different sources, resulting in a series of successful drug concentration gradients in the liquid storage chamber. Then, 24 outlets were connected to 24 single-cell capture devices, which laid the foundation for subsequent cellular resistance research and analysis. The entity map of an integrated microfluidic device based on a multiconcentration gradient and single-cell capture is shown in Fig. S2. The concentration-gradient generator has very high flexibility, with a design of three inlets and a spread-mixing effect of the Tai Chi-spiral mixers. Three concentration gradients can be formed to meet the requirements of a single drug concentration gradient and joint screening of two drugs. Therefore, the mixer can be used to study the possibility of a single drug and multidrug combination and its optimal dosage^37^.

The single-cell capture device consists of five sets of capture matrix columns arranged into a two-dimensional array. Each capture matrix has 180–205 capture units (25 μm height) for single-cell capture. As shown in Fig. 1b, each capture unit consists of two adjacent “H” shaped microstructures for single-cell capture. The two adjacent “H” structures constitute a single-cell capture structural unit. The microstructural capture unit has two minimum pores: the first pore is 2 μm wider than the second pore. Due to the array differences, the spacing of the capture unit decreases. Cell and reagent portals are used for cell suspension and reagent import (Fig. 1c). Single-cell capture and drug stimulation can be achieved using H-shaped capture structures. Other designs of chip dimensions are described in detail in our previous publication^38^.

### Three concentration-gradient formations

**Fig. 2:**
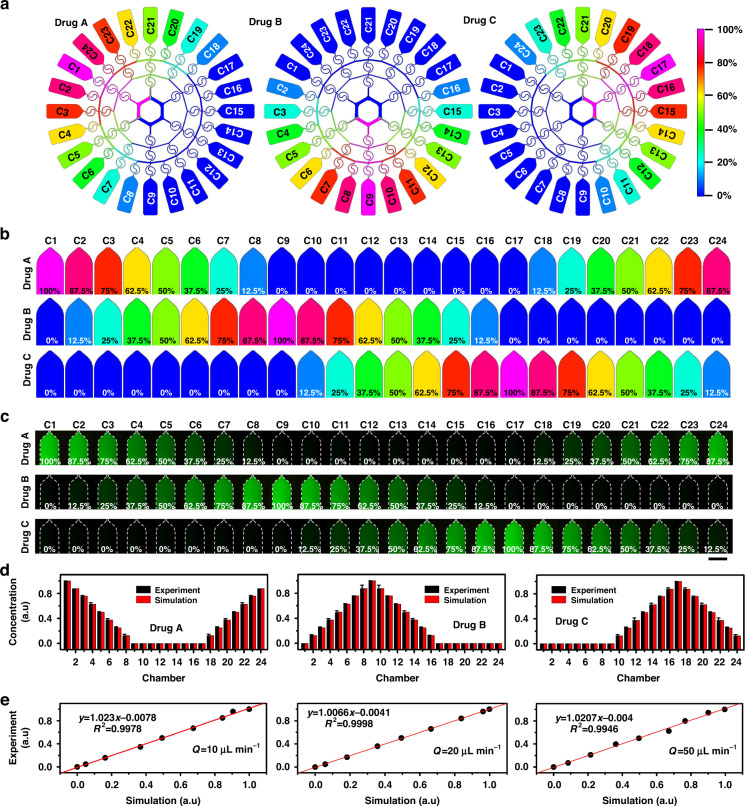
Generation of microfluidic drug concentration gradient under different flow conditions. **a** Simulation imaging of the generation process of the drug concentration gradient in the device. Three inlets were filled with drug A, drug B, and drug C when the flow rate was 10 μL/min. **b** Numerical simulation of drug concentration gradient formation in 24 microchambers. **c** Concentration gradients formed by fluorescence experiments in 24 microchambers. The transport and distribution of substances in the system and the formation of a concentration gradient were fluorescence characterized using luciferin as a model drug. Scale bar: 1200 μm. **d** Fluorescence experiment and computer-simulated concentration value in 24 microchambers. The error bars refer to the standard deviations obtained from ten parallel experiments. **e** Linear relationship of drug concentration in the first nine microchambers by fluorescence experiment and numerical simulation under different flow conditions

To determine whether our device is capable of establishing three concentration gradients, both numerical simulations and fluorescein experiments were performed to explore the distribution of the drug gradients in the 24 microcavities. An inverted fluorescence microscope was used to gather the fluorescein images, and IPP software was then utilized to analyze the images, yielding fluorescein concentration data for each chamber. Finally, the obtained data were processed to obtain the mean and standard deviation. The simulation results showed that three groups of identical drug concentration gradients were formed in the designed device (Fig. 2a). Successive Tai Chi-spiral mixer control and regulation realized a stable mixing state. Accurate drug concentration gradients were steadily fabricated in 24 liquid storage chambers (Fig. 2b). The concentration gradients of drug A were distributed in chambers 1–8 and 18–24; the concentration gradients of drug B were distributed in chambers 2–16; and the concentration gradients of drug C were distributed in chambers 10–24. The simulation percentages of multiple drug concentration gradients were thus generated.

Furthermore, we used fluorescein experimental evaluation to verify the distribution of concentration gradients in the 24 liquid storage chambers at the same flow rate. Luciferin and PBS solution were injected into the chip from three entrances. As shown in Fig. 2c, a series of solutions containing different concentrations of luciferin were also generated in the liquid storage chambers. The experimental results are consistent with the numerical simulation results (Fig. 2d). More importantly, by comparing and analyzing the numerical simulation and fluorescein experiments under three flow conditions, it was found that the concentration difference of each liquid storage chamber was not significant at different flow rates, indicating that our device achieved excellent mixing. The average signal intensity obtained from the fluorescence images showed a good linear relationship with the expected numerical simulation concentration (Fig. 2e). These results demonstrated that three sets of stable and symmetrical concentration gradients could be completely constructed in a velocity-insensitive microfluidic system.

**Fig. 3:**
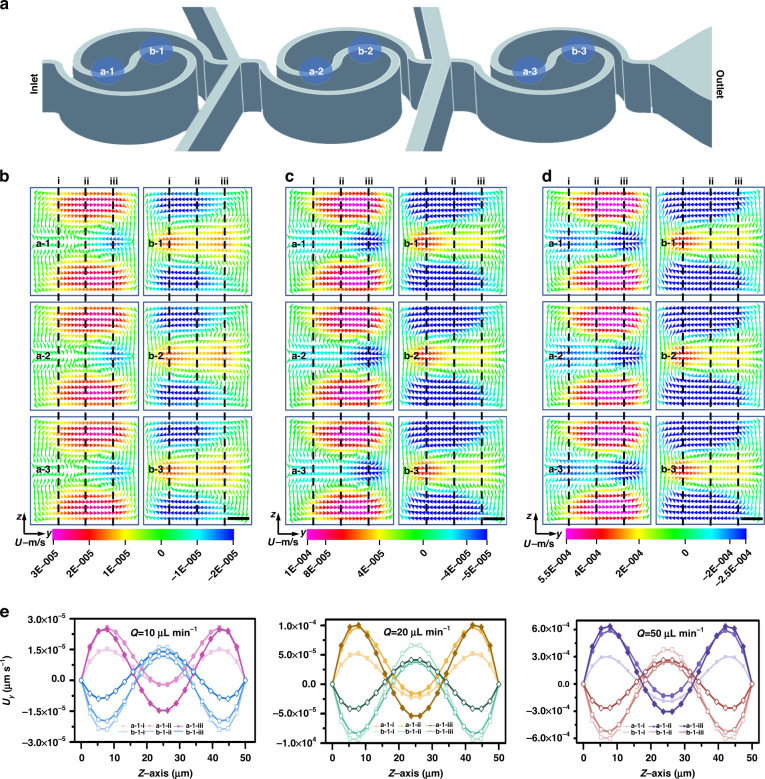
Tai Chi-spiral mixer-induced Dean flow. **a** Sketch diagram of spiral mixer channels. **b**–**d** Dean flow simulation at different positions in the channel when the flow rate was 10 μL/min (**b**), 20 μL/min (**c**), and 50 μL/min (**e**). Scale bar: 10 μm. **e** Quantitative analysis of cross-sectional Dean flow. The results are obtained at the positions shown in the dotted lines of Fig. 3b–d

To further explain the mechanisms and verify the reliability of the above experiments, we further considered the Tai Chi-spiral mixer used in the concentration gradient generator. Many microfluidic applications of fluid manipulation have been developed using superior features of Dean flow in curving channels^40,41^. Due to a fluid momentum disparity between the center and near-wall region of curved channels, a pressure gradient in the radial direction is created, which results in the formation of transverse Dean flow. This Dean flow has revolutionized approaches for sample manipulations, including facilitating mixing, purification, focusing, transport, synthesis, and reactions due to its increased fluidic controllability, affordability, continuousness, and efficacy^37,42^. Dean flow simulation for the two sections of the spiral mixers (Fig. 3a) was carried out. Under different flow conditions, the Dean flow field distribution changes on the upper and lower sides of the spiral mixer channel (Fig. 3b–d). As the flow rate increased, the Dean flow intensity in the fluid velocity field gradually increased (Fig. 3e), and the resulting Dean flow could enhance the solution mixing effect in a relatively short period of time. Additionally, simulation of the velocity field at the S-shaped junction indicated that the Dean vortex direction alternated between anticlockwise and clockwise spirals in succession. The reversal phenomenon further improved the mixing performance, as it facilitated rapid contact and mixing between liquid molecules, resulting from the competition between secondary flow and reaction time. The above results showed that the concentration gradient generator achieved adequate fluid mixing and uniform fluid splitting across a wide range of flow rates. A stable and effective multidrug concentration gradient was successfully achieved, which laid a foundation for subsequent single-cell drug screening based on the formation of three concentration gradients.

### Single-cell drug interaction study

To evaluate the capability of our constructed multiple-concentration platform for single-cell level drug screening, the separate action and combination interactions of anticancer drug A (5-FU) and drug B (DDP) on single tumor cells were explored under the same operating conditions. Then, drug A (with a specific initial concentration of 100 μM) and drug B (with an initial concentration of 10 μM) were routinely applied for chemotherapy^43–45^.

At the same time, a normal medium (without drugs) was introduced into the device from the inlet of drug C. The two anticancer drugs produced three concentration gradients in the fluid storage chambers. In these circumstances, the percentages of 5-FU (drug A) concentrations (0, 12.5, 25, 37.5, 50, 62.5, 75%, 87.5, and 100%) changed from chamber 17 to chamber 1 in ascending order. The percentages of DDP (drug B) concentrations (100, 87.5, 75, 62.5, 50, 37.5, 25, 12.5, and 0%) changed from chamber 9 to chamber 17 in descending order. Single tumor cells from chambers 2 to 8 were treated with different combinations of 5-FU and DDP. Therefore, the ratio of 5-FU and DDP in chambers 2, 3, and 4 was opposite that of chambers 8, 7, and 6. Chamber 17, with a drug-free medium, was used as the control.

**Fig. 4:**
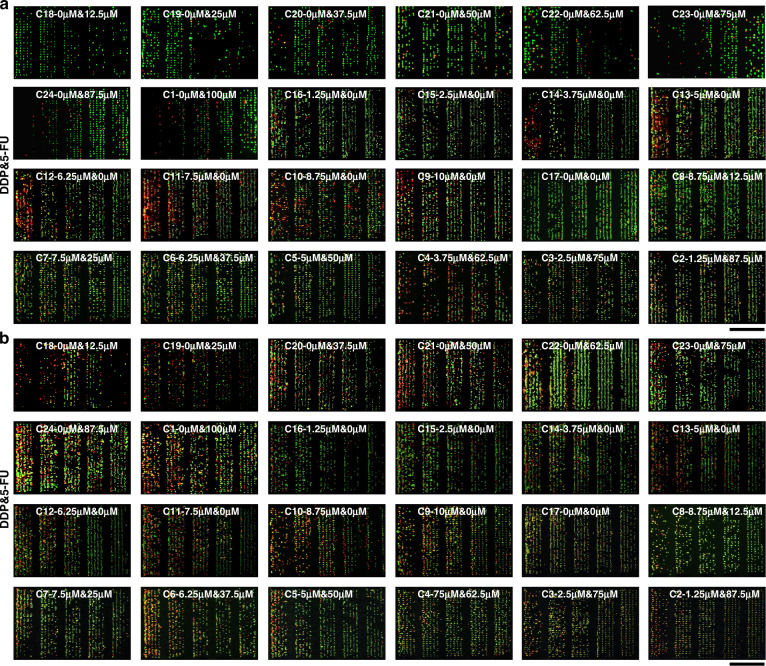
Response of tumor cells in the largest filter units of single-cell capture structures to multiple-gradient dosages of two drugs (5-FU, DDP). **a** Fluorescence images of HepG2 cells were obtained by AO/PI staining after continuous treatment with different concentrations of drugs for 2 h. **b** Fluorescence images of MCF-7 cells were obtained by acridine AO/PI staining after continuous treatment with different concentrations of drugs for 2 h. Scale bar: 800 μm

**Fig. 5:**
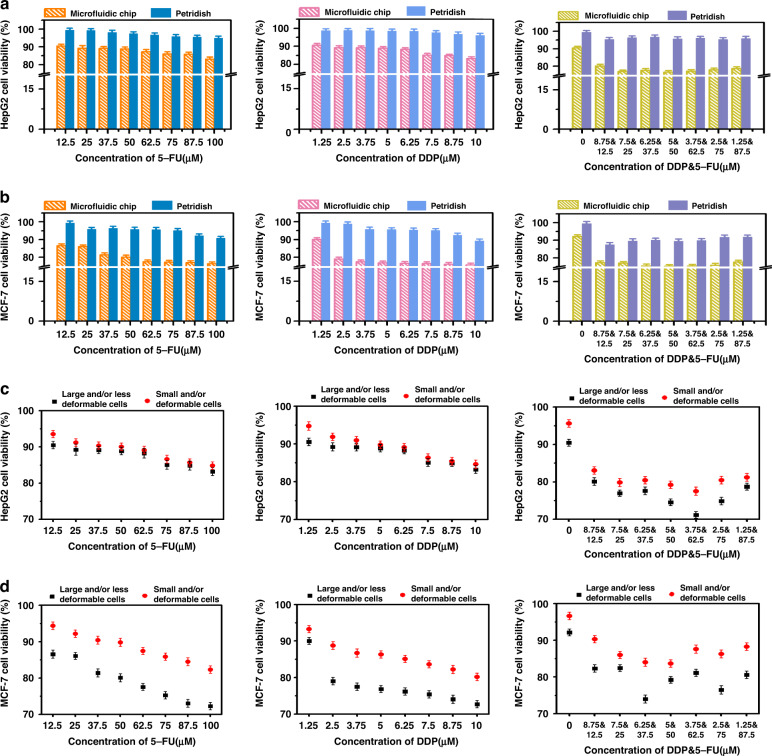
Quantitative comparison of cell viability under multiple-gradient dosages of two drugs (5-FU, DDP). **a**, **b** Quantitative comparison of MCF-7 cell viability (**a**) and HepG2 cell viability (**b**) in single-cell level culture in microarray and Petri dishes. **c**, **d** Quantitative comparison of MCF-7 cell viability (**c**) and HepG2 cell viability (**d**) in the different biomechanical heterogeneity of tumor cells at the single-cell level

Next, a drug-containing medium with different concentrations was applied to HepG2 and MCF-7 cells in the single-cell capture device and in traditional Petri dishes (Fig. 4 and Fig. S3), respectively. The double fluorescence staining technique (acridine orange (AO) and propidium iodide (PI)) was used to evaluate cell viability, with living cells identified as green and dead cells as red. The activity of the cells was monitored after microfluidic single-cell capture. Variations in cell activity were found when different concentrations of the drugs were utilized. The results showed that the survival rate of tumor cells cultured by a single drug (5-FU or DDP) increased with decreasing single drug concentration in Petri dishes and single-cell capture devices (Fig. 5a, b). Cell vitality was negatively correlated with drug dose. The untreated single cells (i.e., the control) in chamber 17 showed normal viability and proliferation within 2 h of culture. When cells were stimulated with the same drug, cell activity at the single-cell level was lower than that in conventional plate cultures. This indicated that there may be cell interactions in traditional plate culture with population effects, which can inhibit the influence of anticancer drugs on cells to a certain extent. The average response of the population is usually obtained in traditional flat culture, which compensates for heterogeneity between cells. However, tumor cells at the single-cell level are not affected by cell interactions and can be effectively studied for their susceptibility to drugs^46–48^.

Furthermore, when the two drugs were combined to act on the cells, the activity of tumor cells in the Petri dishes was lower than that of a single drug, indicating that when combined, the two drugs formed a synergistic effect on cells that was stronger than the effect of a single drug. The obtained results are similar to those previously reported discoveries and clinical trials in which combination therapy showed advantages over monotherapy^46^. Interestingly, this phenomenon can also be found in single-cell capture devices of microfluidic chips when HepG2 cells are observed (Fig. 5a). For MCF-7 cells, the influence of the two drugs on cell viability was noticeable and not indicative of synergy (Fig. 5b). We speculate that this occurred due to continuous infusion of drugs in the chip. The exact explanation for this phenomenon remains unclear and warrants further investigation. This is the first time that multigradient dosing of two drug candidates has been demonstrated at the single-cell level, which offers the potential to effectively screen monotherapeutic regimens and combination therapies.

In addition, to further investigate and analyze the relationship between the biomechanical heterogeneity (such as deformations and size) of tumor cells at the single-cell level and potential drug resistance, we selected individual cells as captured by the smallest filter unit (6–8 μm) and the largest filter unit (14–16 μm) The viability of small and/or “more deformable” and large and/or “less deformable” cells all presented a dose-dependent mode after drug stimulation (Fig. 5c, d). A concentration gradient-dependent increase in the mortality of single cells was observed during the experiment. At multigradient doses of the two drug candidates, small and/or more deformable cells showed higher resistance at the single-cell level than cells with large and/or less deformable characteristics, indicating that the biological heterogeneity of cells was correlated with their drug resistance. That is, tumor cells with a small size or large deformability had stronger drug resistance than tumor cells with a large size or poor deformability. This may occur due to the small size and high degree of deformation of tumor cells, which is related to the high proportion of tumor-initiating cells and high resistance to chemotherapy drugs^47,48^.

Finally, the main manifestations of 5-FU and DDP lethality were DNA synthesis and cell mitosis arrest^45,49^, with the effects weakened for small tumor cells because these cells were predominantly in the G0/G1 phase of their cell cycle. These results indicated that functional gradient-like cell phenotypes in single cells were reconstructed successfully in the microfluidic device, which has advantages over previous microfluidic systems^11,17^. It is also shown that this platform could provide a potential way to study drug screening by exploring tumor cellular heterogeneity at multiple drug gradients while achieving the required stability and high-throughput capability. This development would help facilitate broader biological and preclinical drug explorations, such as screening-based cancer stem cell separation and drug discovery.

## Conclusion

In summary, we developed a simple and efficient multifunctional integrated microfluidic drug screening device based on the single-cell level operation. The device mainly consists of a concentration-gradient drug generator and a single-cell capture array that can be used for multiple purposes. Drug gradients generated by inertial microfluidics at different flow rates can maintain stable and controllable mixing. The single-cell capture array can efficiently separate cells with different sizes and deformability. By using multiple-concentration gradient generators to form different concentration series, the system can be combined with a single-cell capture device to achieve 5-FU and DDP with different doses on HepG2 and MCF-7 cell lines at the level of single-cell biology applicability. Moreover, multifunctional studies of multiconcentration drug-induced tumor responses could be conducted simultaneously in a precisely controlled device. We anticipate that our work can provide a starting point for studying the sensitivity of multiple antineoplastic agents in single cells and for effectively screening monotherapy and combination therapy.

## Supplementary information

Supplementary Information
