# Supplementary material for: Construction of multiple concentration gradients for single-cell level drug screening
Source: Microsyst Nanoeng. 2023 Apr 13;9:46. doi: 10.1038/s41378-023-00516-0 (PMC10102073; doi:10.1038/s41378-023-00516-0)
Supplement: Supplementary file 1 — Supplementary Information [file 41378_2023_516_MOESM1_ESM.docx]

Supplementary Information

**Construction of** **multiple concentration gradients for** **single-cell level drug screening**

Shaofei Shen^a*,1^, Fangjuan Zhang^a,1^, Yali Zhang^a^, Yi Li^a^ , Yanbing Niu^a^, Long Pang^b^*, Jinyi Wang^c^*

*^a^ Shanxi Key Lab for Modernization of TCVM, College of Life Science,* *Shanxi Agricultural University, Taigu, Shanxi 030801, China.*

*^b^ School of Basic Medical Science, Xi'an Medical University, Xi'an,* *Shaanxi 710000, China.*

*^c^ College of Chemistry and Pharmacy, Northwest A&F University, Yangling, Shaanxi 712100, China.*

* Corresponding author. Tel/fax: +86 354 6287205. *E-mail addresses*: shenshaofei@nwafu.edu.cn (S. Shen), [panglong2012@nwsuaf.edu.cn](mailto:panglong2012@nwsuaf.edu.cn) (L. Pang), *jywang@nwsuaf.edu.cn* (J. Wang)*.*

^1^ Contributed equally to this work.

**1. Materials and reagents**

RTV 615 poly(dimethylsiloxane) (PDMS) prepolymer and curing agent were purchased from Momentive Performance Materials (Watford, NY, USA); The surface oxidized silicon wafers were from Shanghai Xiangjing Electronic Technology Co., Ltd (Shanghai, China); AZ 50XT photoresist and developer were from AZ Electronic Materials (Somerville, New Jersey, USA); 5-Fluorouracil (5-FU), Cisplatin (DDP), Fluorescein (FDA), Penicillin-streptomycin stabilized solution was purchased from Sigma-Aldrich (MO, USA); Acridine orange (AO) and propidium iodide (PI) were purchased from Keygen Biotech (Nanjing, China); Dulbecco's modified Eagle medium (DMEM), fetal bovine serum (FBS), trypsin and phosphate buffered saline (PBS) were from Gibco Invitrogen Corporation (California, USA). Fibronectin (FN) comes from Solarbio (Beijing, China). Unless otherwise noted, the analytical reagent grade solvents and other chemicals were purchased from local commercial suppliers. All solutions were prepared with ultrapure water provided by Milli-Q system (Millipore®). MCF-7 cells and HepG2 cells were provided by the Stem Cell Bank of the Chinese Academy of Sciences.

**2. Cell staining and cell viability assay**

Cell viability assessment was performed using a common AO/PI staining protocol. After removing the growth medium and washing with PBS, the AO/PI staining solution (10 μg∙ mL^-1^ each in PBS) was introduced into the chambers at 10 μL∙min^-1^ and the staining process was performed for 10 min at room temperature. Then, PBS was introduced for 10 min as a final rinse. Fluorescence images were taken in each chamber to assess the viability in 5-FU or DDP or 5-FU &DDP gradients. Images taken were compared to control images in each channel. Number of cells were obtained from the analysis of images using count and measure objects of Image-Pro1 Plus 6.0 (Media Cyternetics, Silver Spring, MD). Cell activity was defined as follows:

$\text{C}\text{ell}\text{ }\text{Viability= }\frac{\text{Numeral of liv}\text{ing}\text{ cell}\text{s}}{\text{Numeral of }\text{total}\text{ cell}\text{s}}\text{ ×100\%}$ (1)


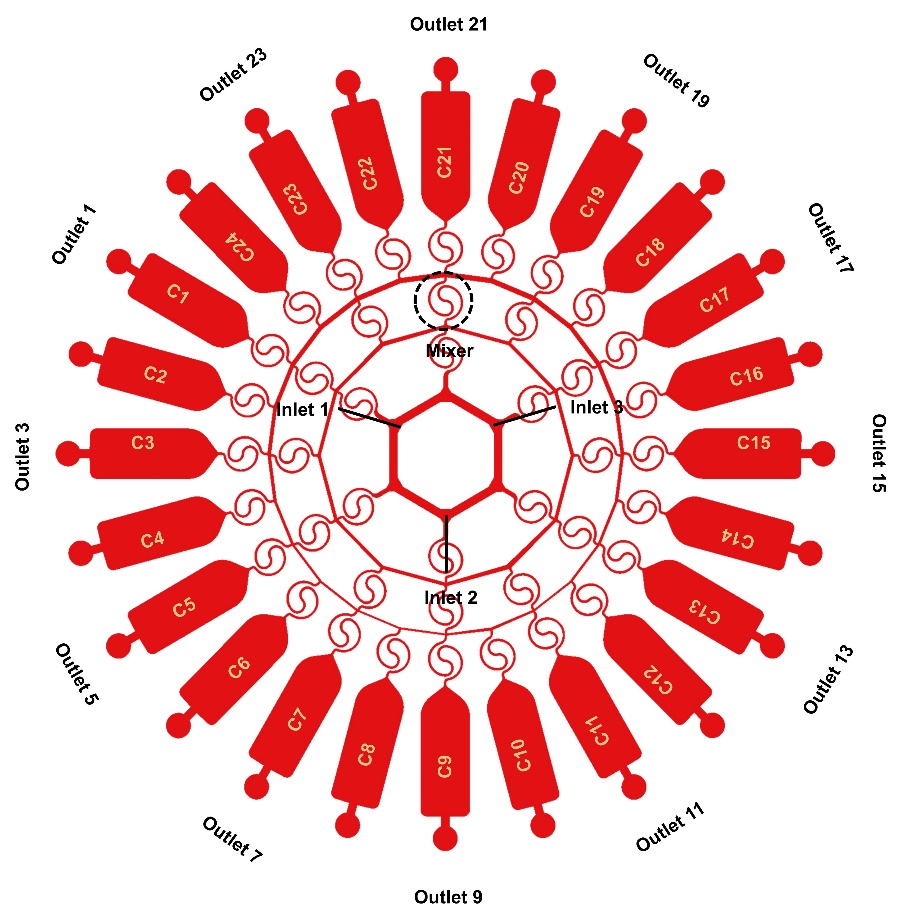


**Figure S1.** The detailed design of a multi-concentration gradient construction device. The height of device is 50μm. The mixer represents our design Tai Chi-spiral mixer. The C1-C24 represent 24 liquid storage chambers.


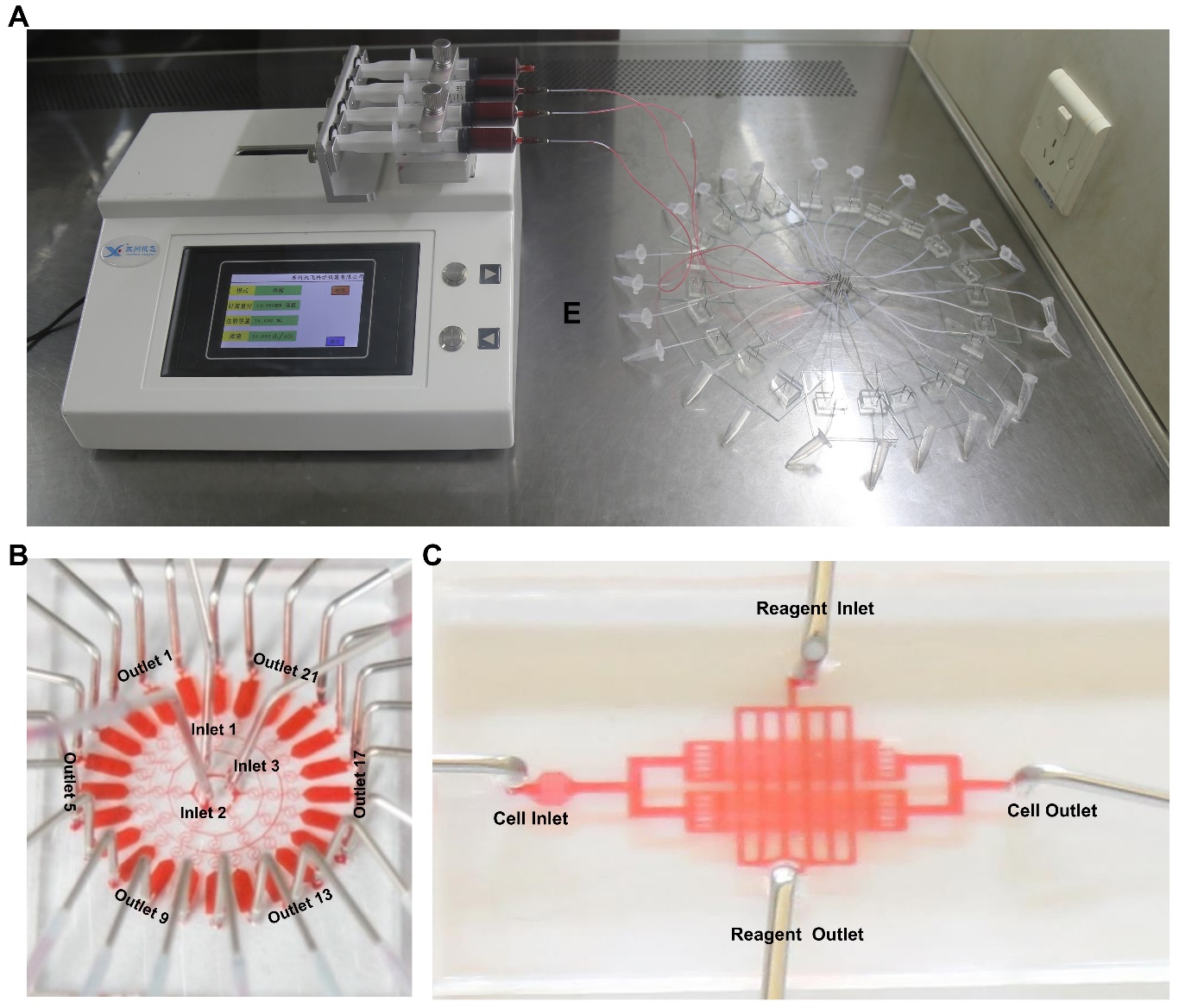


**Figure S2.** (A) The entity map of integrated microfluidic device based on multi-concentration gradient and single-cell capture. (B) Actual diagram for multi-concentration gradient construction device. (C) Actual diagram for single-cell capture device.


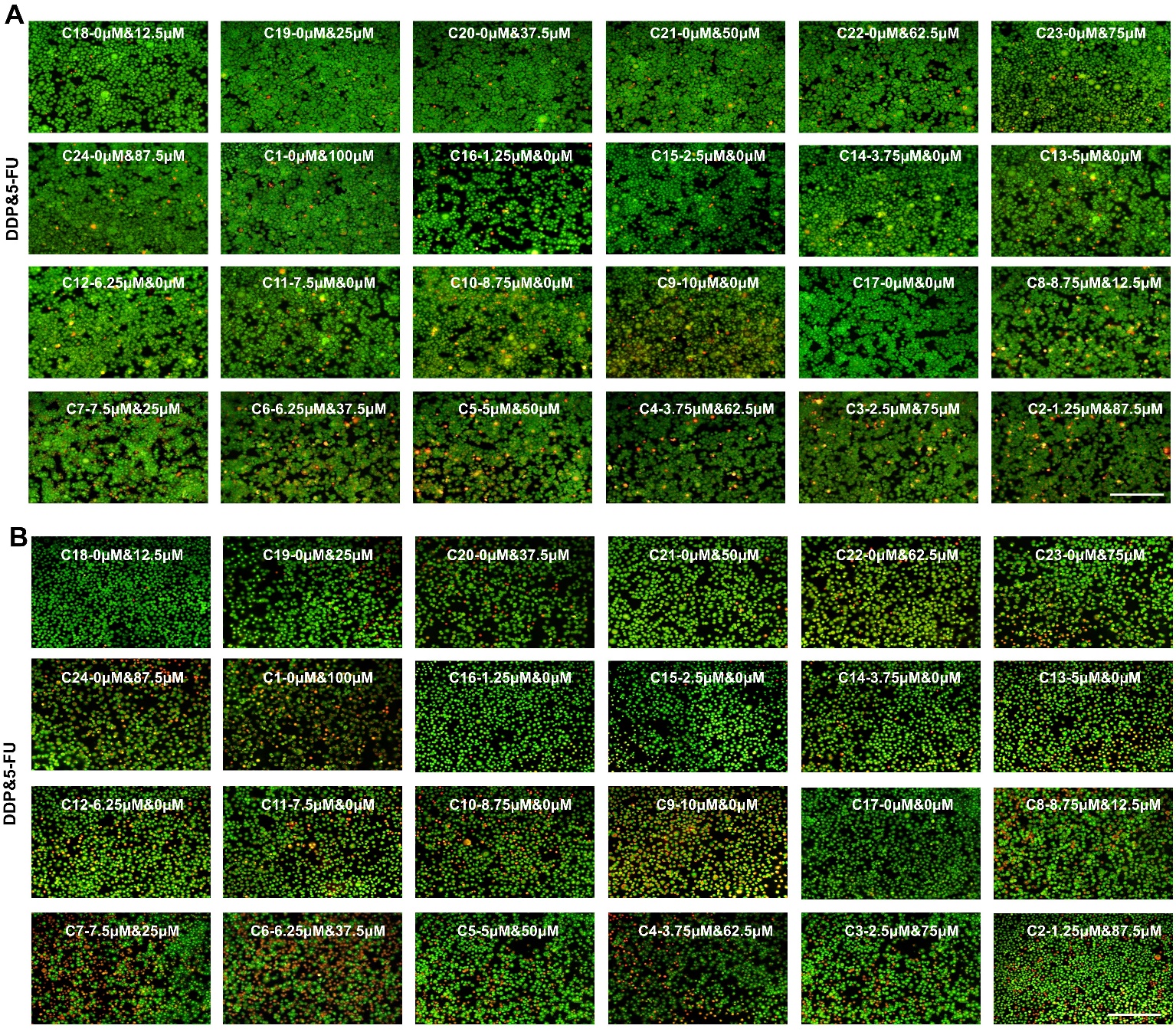


**Figure S3.** Response of tumor cells in Petri dishes to multiple-gradient dosage of two drugs (5-FU, DDP). (A) Fluorescence images of HepG2 cells were obtained by AO/PI staining after continuous treatment with different concentrations of drugs for 2 h. (B) Fluorescence images of MCF-7 cells were obtained by acridine AO/PI staining after continuous treatment with different concentrations of drugs for 2 h. Scale bar: 400 μm.
